# Synthesis, characterization and apoptotic activity of quinazolinone Schiff base derivatives toward MCF-7 cells via intrinsic and extrinsic apoptosis pathways

**DOI:** 10.1038/srep11544

**Published:** 2015-06-25

**Authors:** Maryam Zahedifard, Fadhil Lafta Faraj, Mohammadjavad Paydar, Chung Yeng Looi, Maryam Hajrezaei, Mohadeseh Hasanpourghadi, Behnam Kamalidehghan, Nazia Abdul Majid, Hapipah Mohd Ali, Mahmood Ameen Abdulla

**Affiliations:** 1 Institute of Biological Science, Faculty of Science, University of Malaya, 50603 Kuala Lumpur, Malaysia; 2 Department of Chemistry, Faculty of Science, University of Malaya, 50603 Kuala Lumpur, Malaysia; 3 Department of Pharmacy, Faculty of Medicine, University of Malaya, 50603 Kuala Lumpur, Malaysia; 4 Department of Biomedical Science, Faculty of Medicine, University of Malaya, 50603 Kuala Lumpur, Malaysia

## Abstract

The current study investigated the cytotoxic effect of 3-(5-chloro-2-hydroxybenzylideneamino)-2-(5-chloro-2-hydroxyphenyl)-2,3-dihydroquinazolin-41(H)-one (A) and 3-(5-nitro-2-hydroxybenzylideneamino)-2-(5-nitro-2-hydroxyphenyl)-2,3-dihydroquinazolin-4(1H)-one (B) on MCF-7, MDA-MB-231, MCF-10A and WRL-68 cells. The mechanism involved in apoptosis was assessed to evaluate the possible pathways induced by compound A and B. MTT assay results using A and B showed significant inhibition of MCF-7 cell viability, with IC_50_ values of 3. 27 ± 0.171 and 4.36 ± 0.219 *μ*g/mL, respectively, after a 72 hour treatment period. Compound A and B did not demonstrate significant cytotoxic effects towards MDA-MB-231, WRL-68 and MCF-10A cells. Acute toxicity tests also revealed an absence of toxic effects on mice. Fluorescent microscopic studies confirmed distinct morphological changes (membrane blebbing and chromosome condensation) corresponding to typical apoptotic features in treated MCF-7 cells. Using Cellomics High Content Screening (HCS), we found that compound A and B could trigger the release of cytochrome *c* from mitochondria to the cytosol. The release of cytochrome *c* activated the expression of caspases-9 and then stimulated downstream executioner caspase-3/7. In addition, caspase-8 showed remarkable activity, followed by inhibition of NF-κB activation in A-and B-treated MCF-7 cells. The results indicated that A and B could induce apoptosis via a mechanism that involves either extrinsic or intrinsic pathways.

Aside from being the most common cancer affecting women, breast cancer is also the major cause of death among women globally^1^. Breast cancer represents 31.1% of newly diagnosed cancer cases among women^2^. Based on the latest reports, approximately one million women are diagnosed with breast cancer globally every year^3^. In particular, the occurrence of breast cancer among Malaysian women has surged, with a frequency of 47.4 per 100,000 females, as estimated by the National Cancer Registry Report from 2003–2005^4^. In 2012, The International Agency for Research in Cancer (GLOBOCAN) estimated the rate of breast cancer in Malaysian women as 38.7 per 100,000^5^. Over the past few decades, the number of cases increased significantly because of lifestyle advances and the massive changes in epidemiological properties, such as lower birth and breast-feeding rates^4^.

Cancers are groups of cells that result from a single cell and are characterized by a lack of normal growth regulation known as apoptosis or programmed cell death. This fundamental process plays an important role in the maintenance of tissue homeostasis and the elimination of damaged cells^6^. Therefore, major pharmaceutical companies are focused on apoptosis-based therapy in drug development. Morphological characteristics of apoptotic cells include chromatin condensation, plasma membrane blebbing, cell shrinkage, chromosomal DNA fragmentation and the formation of apoptotic bodies 7,8. The process is primarily activated via receptor-mediated pathways (extrinsic) or mitochondrial mediated signaling pathways (intrinsic), which lead to the activation of caspase-8 and caspase-9, respectively^9^. The intrinsic signaling pathways stimulate apoptosis via the generation of intracellular signals that act directly on targets within the cell through mitochondrial initiated events when cytochrome *c* is released into the cytoplasm^5^,^6^. On the other hand, in the extrinsic signaling pathway, transmembrane death receptors, such as the tumor necrosis factor (TNF) receptor, are closely involved in the initiation of the apoptotic process^10^,^11^. The final pathway of apoptosis, whether extrinsic or intrinsic, is the activation of the execution effector caspases, including caspase-3/6/7^12^,^13^. These caspases activate cytoplasmic endonucleases, which degrade nuclear material, as well as proteases that lead to degradation of the nuclear and cytoskeletal proteins^14^. Apart from caspases, accumulation of excessive ROS will also lead to nuclear DNA damage, followed by disruption of the mitochondrial membrane potential (MMP) and release of cytochrome c into the cytosol^15^.

Recently, substantial research has been performed with quinazolinones and their derivatives to discover novel applications in medical chemotherapy^16^. The quinazoline nucleus and its derivatives are a class of heterocyclic compounds that are considered to be the basic framework of biologically active compounds that exist in a number of drug molecules and biologically active compounds. They have attracted the attention of biologists and medicinal chemists because they exhibit various types of pharmacological activities, such as: anticancer^17^, antioxidant^18^, antiviral^19^, anticonvulsant^20^, anti-inflammatory^21^, antitubercular^22^, anti-HIV^23^, and so on. Many efforts have been made by chemists to modify the quinazoline ring for the development of biological, pharmaceutical and clinical compounds. As a continuation of previous efforts, researchers now aim to synthesize and develop new active quinazolines by different synthetic routes to obtain a wide range of biological activities.

Quinazolinones and their derivatives have been found to provide several benefits over the agents that are clinically used^24^. Accumulating evidence shows that quinazolinones is closely connected to the anti-cancer therapies^25^,^26^. For instance, quinazolinones derivatives were proved substantial in treating human leukemia than the conventional agents^27^. Recent studies have shown the significant effect of quinazolinones derivatives against breast cancer cell lines^28^,^29^,^30^. ZD1839 is a quinazoline derivative that selectively inhibits the EGFR tyrosine kinase activity and is currently used for breast cancer patients^31^. Furthermore, numerous studies have been done to assess the pharmacokinetics and toxicity of new quinazoline-based compounds in different animal model to demonstrate the safe nature of their synthesized compounds^32^,^33^. In this study, we synthesized two new quinazolinones and evaluated their anticancer potential against human breast cancer MCF-7 cells. Using the same cell-line, we investigated the cell death mechanism underlying this activity.

## Results

### IR study

IR spectra results from the compounds (A and B) show two absorption bands for a hydroxyl group at 3368 cm^−1^ for compound (A) and at 3365 cm^−1^ for compound (B). Moreover, broad absorption bands located at 3263 cm^−1^ for compound (A) and at 3081 cm^−1^ for compound (B) are attributed to NH of the quinazoline ring. In addition, absorption bands for an amide group for compounds (A and B) appeared at 1662 cm^−1^ and 1649 cm^−1^, respectively. Two sharp absorption bands were located at 1591 cm^−1^ for compound (A) and at 1588 cm^−1^ for compound (B), which were assigned to an azomethine group. Both of the absorption bands for the NH quinazoline ring and azomethine group are evident for the formation of both synthesized compounds.

### ^1^H and ^13^C NMR studies

**Figure 1:**
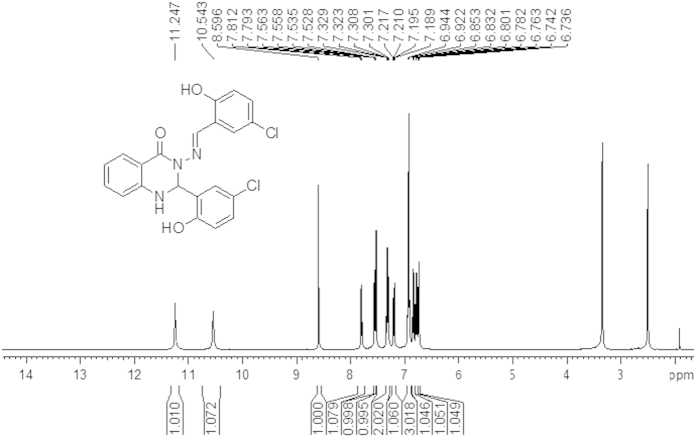
^1^H NMR spectrum of compound (A) in (400 MHz, DMSO-d6).

**Figure 2:**
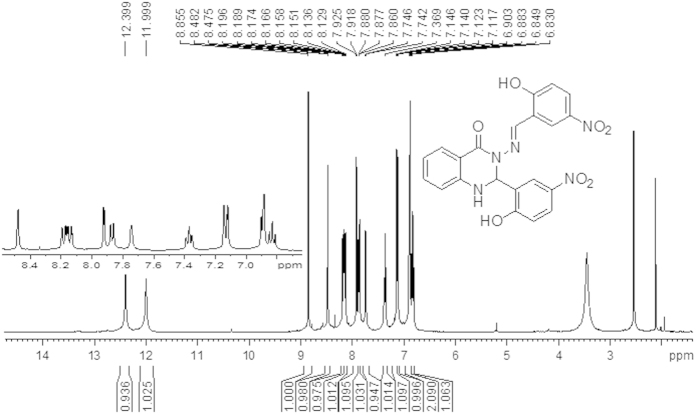
^1^H NMR spectrum of a compound (B) in (400 MHz, DMSO-d6).

The ^1^H and ^13^C NMR results were obtained to allow a more global view of the successful formation of both compounds (A and B). Figures 1 and 2 show two sharp singlet signals at 11.24 ppm and 10.54 ppm for compound (A), respectively, and 12.40 ppm and 12.00 ppm for compound (B), respectively, which are assigned to (O–*H*) groups. In addition, two singlet signals were observed at 8.60 ppm for compound (A) and 8.55 ppm for compound (B) that belong to the C*H*=N azomethine group. Signals for the N*H* of the quinazoline ring from both compounds (A and B) were observed as doublet signals at 7.55 ppm with *J* = 2.24 Hz for compound (A) and at 8.48 ppm with *J* = 2.81 Hz for compound (B). Another two doublet signals were also observed at 6.73 ppm with *J* = 2.25 Hz for compound (A) and at 7.7 4 ppm with *J* = 1.73 Hz for compound (B) that were assigned to protons of the C*H* of the quinazoline ring. The other ten signals were observed between 7.81 ppm to 6.78 ppm for compound (A) and between 8.18 to 6.83 ppm for compound (B) and were attributed to protons of aromatic rings. All of these signals were in good agreement with the number of protons in the proposed structure, which confirmed the formation of the synthesized compounds.

**Figure 3:**
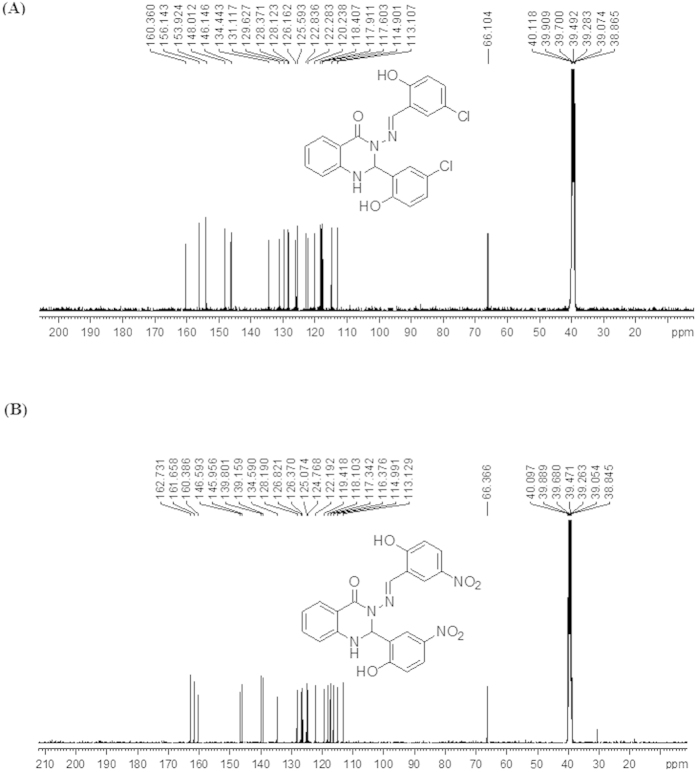
^13^C NMR spectrum of the compounds (A and B ) in (100 MHz, DMSO-d6).

^1^H NMR results were confirmed by ^13^C NMR results. Figure 3 shows a singlet carbon signal of the amide group N-*C*=O of compounds (A and B) that appeared at 160.36 ppm and at 162.73 ppm, respectively. A singlet signal appeared at 148.01 ppm for compound (A) and 146.59 ppm for compound (B) that were attributed to an azomethine group -N=*C*H-, while a carbon atom of the quinazoline ring was observed at 66.10 ppm for compound (A) and 66.36 ppm for compound (B). Other signals in the compound belong to carbon atoms of aromatic rings. As a result, the ^13^C NMR spectrum showed twenty-one resonance signals for each compound (A and B), which were in good agreement with the number of carbon atoms in the proposed structures, thus confirming our ^1^H NMR results.

### Crystallography study

**Figure 4:**
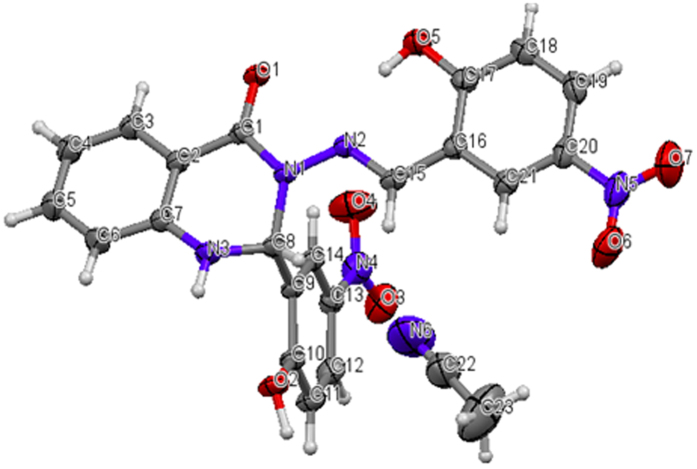
Crystal structure of compound (B).

Figure 4 shows the X-ray crystal structure of compound (B), which consists of two coplanar 2-hydroxy-5-nitrophenyl groups bonded to a distorted (envelope) dihydroquinazoline. There is a significant twist between dihydroquinazoline-4(1*H*)-one and the attached 2-hydroxy-5-nitrophenyl as a result of the torsion angle of the methylene amine linkage. The compound consists of two hydrogen bonds, one is intra-molecular N2…H3A with a distance of 1.874 and the other is intermolecular O4…H3N with distance of 2.062 between the dihydroquinazoline and the solvated acetonitrile (CDCN). All of the X-ray crystallographic data are provided in Table 1.

### Anticancer evaluation of quinazoline Schiff bases

#### MTT cell viability test

An MTT cytotoxicity assay was performed to determine the anti-proliferative effect of both compounds on MCF-7 and MDA-MB-231 cancer cells. The results showed that compounds A and B significantly inhibited the proliferation of MCF-7 cells; however, they did not show remarkable effect towards MDA-MB-231 cancer cells. Meanwhile, they exhibited no suppressive activity against human normal MCF-10A breast cells and normal WRL-68 hepatic cells compared to the IC_50_ value of compounds used on MCF-7 cells. In this assay, the IC_50_ value of doxorubicin was also recorded as a positive control (Table 2).

### LDH cytotoxicity test

**Figure 5:**
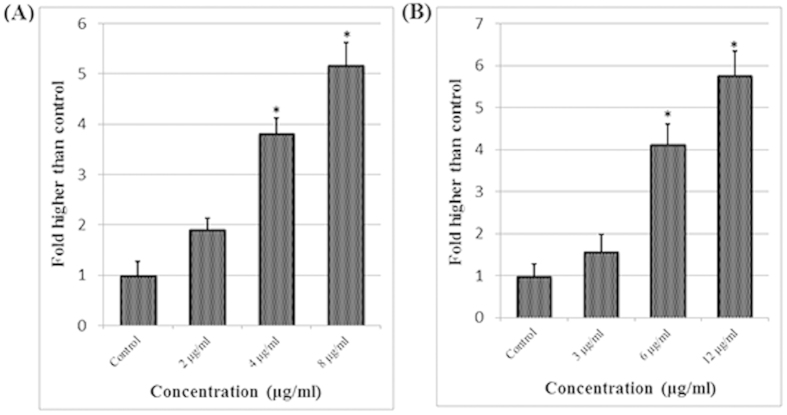
Lactate dehydrogenase (LDH) assay. The LDH release assay revealed significant cytotoxicity of quinazolinone-based compounds on MCF-7 cells at 4–8 μg/mL of compound A and at 6–12 μg/mL of compound B.

Lactate dehydrogenase (LDH) release in the medium is an enzymatic indicator that illustrates the loss of membrane integrity, apoptosis, or necrosis of a cell. The cytotoxic effect of the Quinazolinone-based compounds was also assessed by lactate dehydrogenase (LDH) release on treated MCF-7 cells with different concentrations after 48 hours of incubation. Both compounds induced a significant elevation of LDH release in treated cells compared to control cells (Fig. 5), indicating a significant cytotoxic effect at 4 and 8 μg/mL of A and at 6 and 12 μg/mL of B.

### Morphological assessment by AO/PI double staining

**Figure 6:**
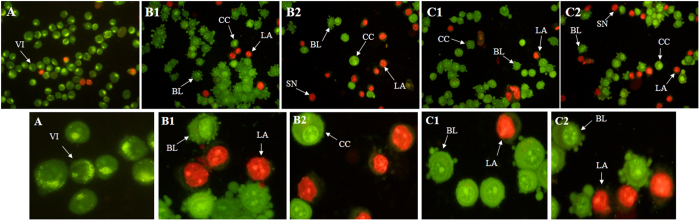
Fluorescent micrographs of AO/PI-double-stained MCF-7 cells. (A) Untreated MCF-7 cells exhibit normal structures. (B1) and (C1) Early apoptosis features, namely, blebbing and chromatin condensation as well as late apoptotic cells were detected after 24 hours of treatment with (A) and (B). (B2) and (C2) Late apoptosis and secondary necrosis were observed after 48 h treatment with (A) and (B), respectively (magnification: 200×). VI: Viable cells; CC: Chromatin condensation; BL: Blebbing of the cell membrane; LA: Late apoptosis; SN: Secondary necrosis.

The AO/PI double staining assay is based on the emission of green and orange fluorescent wavelengths. As shown in Fig. 6, the untreated MCF-7 cells displayed green healthy intact nuclei. After 48 hours of incubation, we detected membrane blebbing and chromatin condensation, which are reflective of early apoptotic actions. These features were more distinct at 72 hours of treatment, which correlated with late apoptosis (the presence of a reddish-orange color) due to the PI-positive band of denatured DNA. Additionally, at an advanced stage of incubation, the existence of secondary necrosis was obvious, as the prolonged incubation of treated MCF-7 cells might trigger secondary necrosis following late apoptosis as evidenced by intense reddish color.

### Generation of Reactive Oxygen Species (ROS)

**Figure 7:**
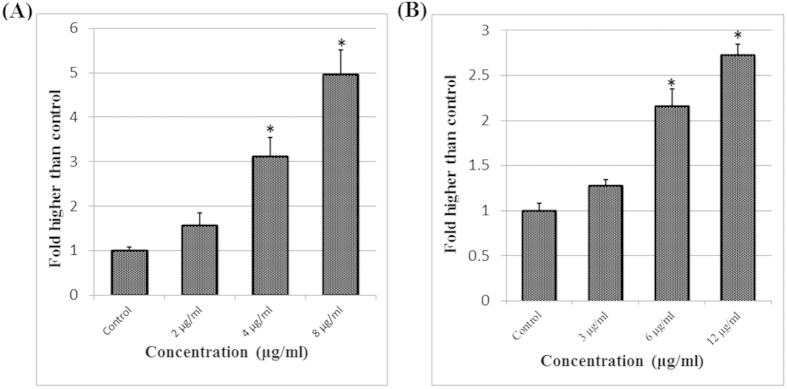
Effect of compound A and B on the generation of ROS. **(A)** The level of ROS was significantly elevated at 4–8 μg/mL concentrations of compound A and **(B**) at 6–12 μg/mL concentrations of compound B.

The production of ROS leads to disturbed homeostasis in the enzyme system of ROS scavenging antioxidants. Hence, the level of ROS was evaluated in the treated MCF-7 cells with different concentrations of A and B. As shown in Fig. 7, exposure to the quinazolinone-based compounds after 24 hours of incubation produced the noteworthy generation of ROS in A- and B-treated MCF-7 cells at doses of 4–8 and 6–12 μg/mL, respectively.

### Mitochondria-initiated events induced by quinazolinone-based compounds

**Figure 8:**
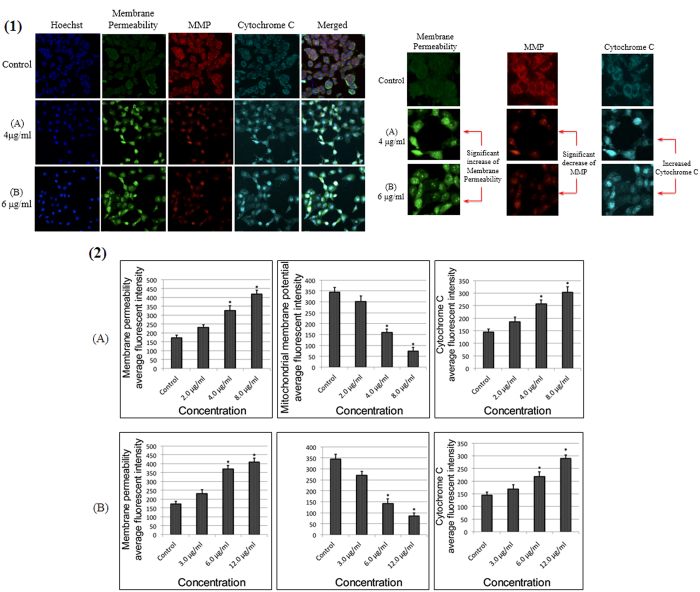
Effects of the Quinazoline Schiff bases on membrane permeability, mitochondrial membrane potential (MMP) and cytochrome c release. (1) Representative images of MCF-7 cells treated with medium alone and compounds (A and B) at 4 and 6 μg/mL, respectively, and stained with Hoechst 33342 for nuclei, cytochrome c, membrane permeability and MMP dyes. Both compounds induced a noteworthy elevation in membrane permeability and cytochrome c release and a marked reduction in mitochondrial membrane potential (magnification: 200×). (2) Representative bar charts indicating dose-dependent increases in cell permeability, reduced MMP and increased cytochrome c release in A- and B-treated MCF-7 cells.

An MMP fluorescent probe was used to analyze mitochondrial function. MMP dye intensely stained the untreated MCF-7 cells; however, the cells treated with the compounds were not stained after 24 hours of incubation. The decline in MMP fluorescent intensities indicated that the MMP is reduced in the A- and B- treated cells at 4–8 and 6–12 μg/mL, respectively. At the same concentrations, a remarkable increase in cell membrane permeability was also detected after 24 hours of exposure in MCF-7 cells to the quinazolinone-based compounds. In addition, A and B significantly activated the translocation of cytochrome *c* from mitochondria to the cytosol compared to the control cells (Fig. 8).

### Evaluation of Caspase-3/7,-8 and -9 Activities

**Figure 9:**
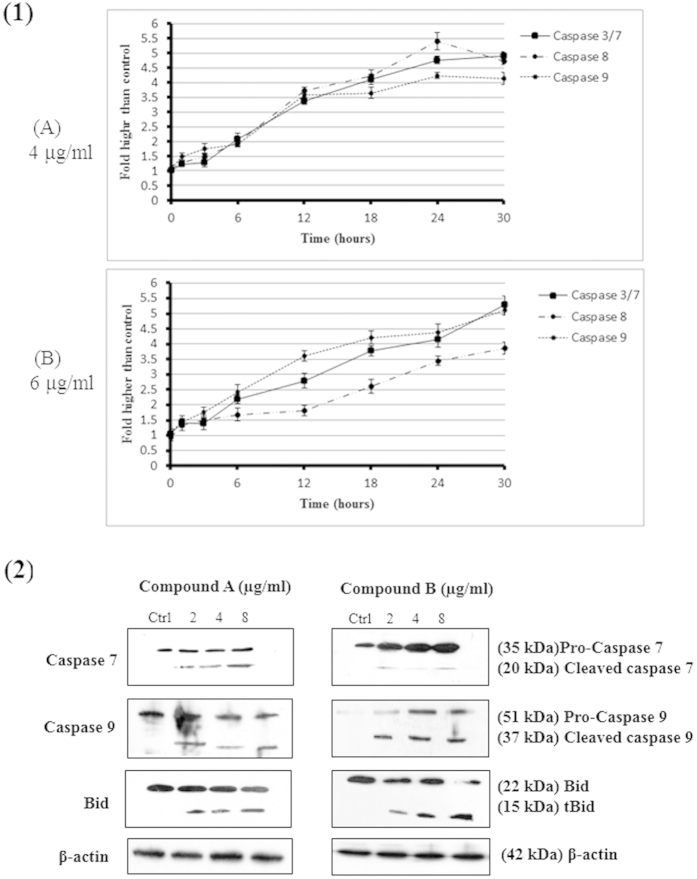
(1) Relative luminescence time-dependent expression of caspases 3/7, -8 and -9. MCF-7 cells treated with A and B at concentrations of 4 and 6 μg/mL, respectively, after 24 hours of incubation showed significant expression of caspases 3/7, -8 and -9. **(2) Western blot analysis of A- and B-treated MCF-7 cells.** Cells were treated with compounds for 24 hours before being lysed and subjected to separation by sodium dodecyl sulfate polyacrylamide gel electrophoresis. Proteins were then transferred to a membrane and probed with antibodies against caspase 7, -9 and Bid. The membrane was reprobed with anti-β-actin antibody as the loading control. The band densities of treated samples were normalized to the control. The results revealed significant activation of caspases -7, -9 and Bid.

Induction of apoptosis via either intrinsic or extrinsic pathways is precisely mediated by caspase cascade events. In this experiment, the bioluminescent intensities of respective caspases indicated that their activities were measured time-dependently in MCF-7 cells treated with IC_50_ concentrations of A and B for 24 hours of treatment. As shown in Fig. 9, a remarkable increase in the caspase -8, -9 and -3/7 activities was quantified in A- and -B treated cells at 4 and 6 μg/mL, respectively. After 12 and 24 hours of incubation, caspase -8, -9 and -3/7 demonstrated noteworthy activation. Furthermore, we detected that the expression levels of caspase -7,-9 and Bid proteins (proapoptotic proteins) were significantly increased in a concentration-dependent manner using western blot analysis (Fig. 9).

### NF-κB Translocation

**Figure 10:**
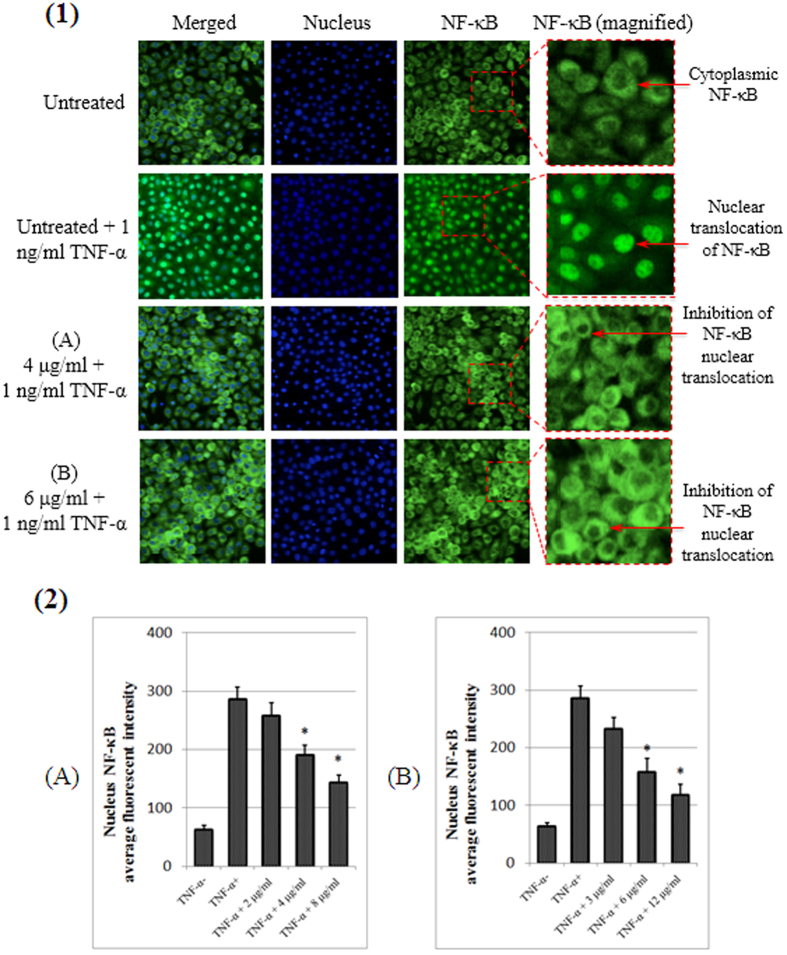
NF-κB translocation. (1) Photographs of the intracellular targets of stained MCF-7 cells that were exposed to A and B at concentrations of 4 and 6 μg/mL, respectively, for 3 hours and then stimulated for 30 minutes with 1 ng/ml TNF-α (NF-κB activation). (2) Representative bar chart showing translocation of TNF-α induced NF-kB in MCF-7 cells at different concentrations.

The inhibition of apoptosis and cell proliferation is closely associated with the activation of NF-κB. Hence, we also tested compounds to evaluate the inhibitory effects against NF-κB translocation from the cytoplasm to the nucleus activated by TNF-α. The results obtained in this research revealed the inhibitory effect of both compounds on TNF-α-induced NF-κB translocation in a dose-dependent manner with a significant inhibition at 4 and 6 μg/ml of A and B, respectively (Fig. 10).

### Acute toxicity

**Figure 11:**
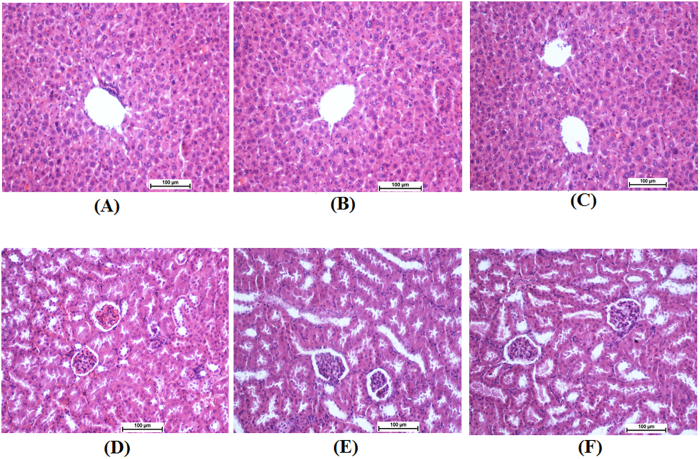
Histological sections in the acute toxicity test (H&E staining, 20x). Histological sections of liver (first row) and kidney (second row). Untreated mice (control group) received 5 mL/kg vehicle (5% Tween 20) ((A) and (D)). Animals treated with 250 mg/kg are ((B) and (E)) and ((C) and (F)) for compounds A and B, respectively. There were no significant differences in the structures of the liver or kidneys between the treated and untreated group.

For the acute toxicity study, mice were treated with A and B at single doses of 250 mg/kg. After 14 days, all animals survived the treatment period. No physical or abnormal changes was observed in the skin, fur, eyes, mucus membranes, tremors, salivation, behavior patterns, or sleep patterns. Kidney and liver biochemical analyses were reported as normal (Tables 3 and 4), and no differences were observed in kidney and liver tissue histopathology analysis between A- and B-treated mice compared to the normal control group (Fig. 11).

## Discussion

Although there are a wide variety of signals and stimuli that can trigger apoptosis, chemotherapy still offers the most effective approach to treat cancer by inducing apoptosis in cells with fewer side effects and higher efficiency. In the current work, we synthesized two quinazolinone Schiff bases and characterized them by IR spectra^34^, ^1^H and ^13^C NMR spectra^35^, and crystallographic study^36^,^37^. Subsequently, the anti-cancer potential and the underlying mechanisms of compounds A and B were investigated in MCF-7, a human breast cancer cell-line. Most approaches applied in cancer therapy, such as chemotherapy and radiation therapy, are likely to be affected by the apoptotic activities of tumor cells, leading to apparent therapeutic implications. Many morphological and biochemical changes are associated with cell apoptosis, which include nuclear fragmentation, mitochondrial potential change, caspase activation, and so on^30^,^38^. In our study, the release of cytochrome *c* from mitochondria into the cytosol and the activation of caspase cascades were observed in A- and B-treated MCF-7 cells. Furthermore, the generation of ROS and the remarkable inhibition of nuclear factor-kappa beta (NF-κB) translocation from the cytoplasm to the nucleus triggered by tumor necrosis factor alpha (TNF-α) were also detected.

MTT is a common assay used to evaluate the cytotoxic effect of new substances on cancer cells, which was first described by Mosmann in 1983 in the detection of mammalian cell survival and proliferation^39^. The MTT results obtained in this experiment revealed that the new quinazolinone Schiff bases selectively inhibited MCF-7 cancer cell viability without affecting breast cancer cells MDA-MB-231 and the normal cells (MCF 10A and WRL-68). The apoptotic effect of these compounds were further established using microscopic analysis by AO/PI staining, which showed distinctive morphological changes associated with typical apoptosis features, such as chromatin condensation and membrane blebbing^30^,^38^.

ROS and mitochondria play an important role in the stimulation of apoptosis, which is known as the intrinsic signaling pathway^11^,^15^. ROS are responsible for the oxidation of mitochondrial pores, leading to cytochrome *c* release due to the disruption of the mitochondrial membrane potential, one of the early events leading towards irreversible apoptosis^30^,^40^. Treatment with A and B produced a significant amount of ROS and consequently caused the disruption of the mitochondrial membrane potential. We also found that cytochrome *c,* a key apoptotic protein, was released from mitochondria, resulting in the full activation of the initiation caspase -9 and execution caspase-7^38^. These results were later confirmed by western blot analysis where the expression of cleaved caspase-7, -9 and Bid proteins were significantly increased in a dose-dependent manner^41^,^42^. Moreover, activation of caspase-8 was also observed in treated cells, which is closely related with apoptosis signaling through the extrinsic pathway^43^. In many cases, caspase-8 could be linked to the mitochondrial pathways by cleavage of the Bcl2 family members Bid to tBid^44^. Aside from Bcl2 family members, NF-κB can also act as an inhibitor of apoptosis and play a significant role in the survival of tumors. In addition, the suppressive activity of NF-κB is closely associated with the activation of the extrinsic apoptosis pathway in tumor cells^41^,^45^. Hence, we assessed both compounds for their inhibitory effects against NF-κB translocation from the cytoplasm to the nucleus and found that A and B could induce apoptosis via suppression of NF-κB as well. Administration of 250 mg/kg of compounds in mice showed safe nature of compounds without any mortality or toxicity effects on liver and kidney examined by histological and biochemical studies^30^.

In conclusion, we have established the structures of synthesized quinazolinone Schiff bases A and B by elemental analysis, spectroscopic techniques and X-ray diffraction studies. Furthermore, our results showed that these compounds induced apoptotic cell death in tumor cells. MCF7 cells treated with A and B triggered apoptosis by disrupting MMP, which caused cytochrome *c* release from mitochondria to the cytosol. Cytochrome c subsequently activates caspase 9 and downstream executioner caspase-3/7, leading to apoptotic changes. Moreover, these compounds induced apoptosis via the extrinsic pathway, which involves caspases (caspase-8) and the inhibition of NF-κB translocation from the cytoplasm to the nucleus.

## Methods

### Reagent and chemicals

All chemicals and solvents used for the synthesis of the compounds (A and B) were obtained from Merck and Sigma-Aldrich. Melting points of the synthesized compounds were determined by an open capillary melting point apparatus and are uncorrected. The purity of the compounds was checked using pre-coated TLC plates from MERCK (60F254) using hexane:ethyl acetate 4:1 as an eluent. The developed chromatography plates were visualized under UV-Vis at 254 nm. Infrared spectra were performed by using a Perkin Elmer spectrum 4000-400 FT.IR / FT-FIR Spectrometer. ^1^H and ^13^C NMR spectra were recorded on an AVN Bruker 400 FT–NMR system. Tetramethylsilane TMS was used as an internal standard, deuterated DMSO was used as a solvent for the NMR spectrophotometer and elemental analysis (CHNS) was performed on an elemental analyzer Perkin Elmer CHNS/O 2400 series II.

### General procedure for the synthesis of quinazoline Schiff bases

**Figure 12:**
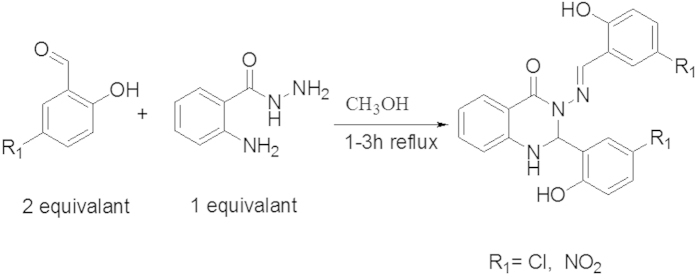
The synthetic pathway of the compounds (A and B).

Both compounds (A and B) were synthesized by using a similar procedure as previously described, with a few modifications^46^,^47^ (Fig. 12). One equivalent of aminobenzhydrazide (2.5 mmol) was dissolved in 50 ml of ethanol and two equivalents of substituted aromatic salicyaldehyde (5.0 mmol) were added to this solution in the presence of glacial acetic acid as a catalyst at 50–60 °C. The reaction mixture was refluxed for 2–3 hours. The yellow precipitates of the compounds (A and B) were formed during the reactions. Three quarters of the solvent was evaporated and the precipitate was filtrated and washed with cold ethanol and then dried in an oven. The purity of the compounds was checked by TLC. The chemical structures of the synthesized compounds have been characterized and confirmed by elemental analysis (CHN) and spectroscopic techniques such as FT-IR, ^1^H, ^13^C NMR spectroscopy and X-ray crystallography.

### Synthesis

#### Synthesis of 3-[(5-Chloro-2-hydroxy-benzylidene)-amino]-2-(5-chloro-2-hydroxy-phenyl)-2,3-dihydro-1H-quinazoline-4-one (A)

**Figure 13:**
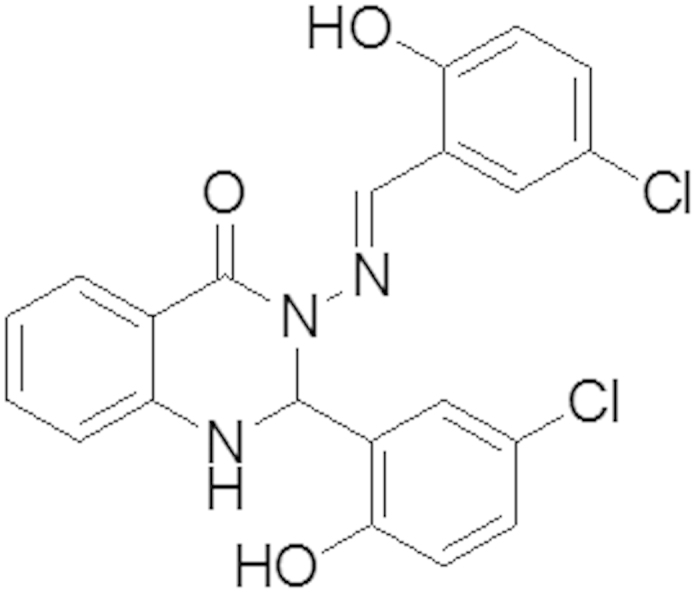
The chemical structure of Compound (A).

As shown in Fig. 13, 2-aminobenzyhydrazide (0.755 g, 5 mmol) was reacted with 2-hydroxy-5-Chloro benzaldehyde (156.57 g, 10 mmol) according to the above-mentioned procedure. Yield: (2.2 g, 97%), m.p 235–237 °C, Anal. Calc. For C_21_H_15_N_3_O_3_Cl_2_ (428.27): C, 58.89, H, 3.53, N, 9.81. Found: C, 58.759, H, 3.146, N, 9.702. IR: 3368 v (O-H), 3263 v (N-H), 1662 v (NC = O), 1591 v (N = CH), 1295 v (N-N), 1158 v (C-N), 1113 (C-O), 820 v (C-Cl). ^1^H NMR (DMSO-*d*_*6*_/TMS, ppm) δ = 11.24 (s, H,O*H* ), 10.54 (s, H,O*H* ), 8.60 (s, 1H, C*H* = N), 7.81 (d, 1H, *Ar*-*H*), 7.55 (d, 1H, *J* = 2.24 Hz, N*H* quinazoline ring), 7.53 (d, 1H, *Ar-H*), 7.32 (dt, 2H, *Ar*-*H*), 7.21 (dd,1H, *J* = 2.56 Hz, *J* = 8.63 Hz, *Ar*-H), 6.94 (d,3H, *Ar*-H ), 6.85 (d, 1H, *Ar*-H), 6.78 (t, 1H, *Ar*-H), 6.73 (d, 1H, *J* = 2.25, C*H* quinazoline ring).^13^C NMR (100 MHz, DMSO-*d*_*6*_/TMS, δ in ppm) δ = 160.36 (N-*C* = O), 156.14 and 153.92 (*A*r-OH), 148.01 (N = *C*H), 146.14, 134.44, 131.11, 129.62, 128.37, 128.12, 126.16, 125.59, 122.83, 122.28, 120.23, 118.40, 114.90 and 113.10 (*A*r-H), 117.91 (*Ar*-Cl), 117.60 (*Ar*-Cl) and 66.10 (*C*H quinazoline ring).

#### Synthesis of 3-(-5-nitro-2-hydroxybenzylideneamino)-2(-5-nitro-2-hydroxyphenyl)-2,3-dihydroquinazoline-4(1H)-one (B)

**Figure 14:**
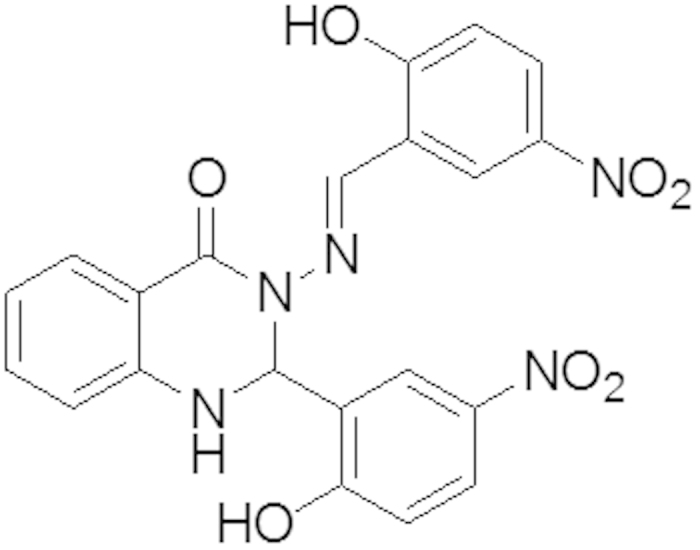
The chemical structure of Compound (B).

As shown in Fig. 14, 2-Aminobenzoyhydrazide (0.755, 5 mmol) and 2-hydroxy-5-nitrobenzaldehyde (1.67 g, 10 mol) react according to the above procedure. Yield: (2.2 g, 96%), mp 250–252 °C. Anal. Calc. For C_21_H_15_N_5_O_7_ (449.37): C, 56.13 H, 3.36, N, 15.58. Found: C, 55.318, H, 3.075, N, 15.299. IR: 3365 v (O-H), 3081 v (N-H), 1649 v (NC = O), 1588 v (N=CH), 1282 v (N-N), 1151 v (C-N), 1107 (C-O), 1333 v (C-NO_2_). ^1^H NMR (DMSO-*d6*/TMS, δ in ppm): δ = 12.39 (s, H, *OH*), and 11.99 (s, H, *OH*), 8.85 (s, 1H, N=C*H*), 8.48 (s, 1H, *J* = 2.81 Hz, N*H* quinazoline ring), 8.18 (dd, *J* = 2.78 Hz, *J* = 9.15 Hz, *Ar-H*), 8.14 (dd, 1H, *J* = 2.71 Hz, *J* = 9.00 Hz, *Ar-H*), 7.92 (d, 1H, *J* = 2.72 Hz, *Ar-H*), 7.86 (dd, 1H, *J* = 1.00 Hz, *J* = 7.80 Hz, Ar-*H*), 7.74(d, 1H, *J* = 1.73 Hz, C*H* quinazoline ring), 7.36 (t, 1H, *Ar-H*), 7.14 (d, 1H, *J* = 2.22 Hz, Ar-*H*), 7.12 (d, 1H, *J* = 2.31 Hz, *Ar-H*), 6.89 (d, 2H, *Ar-H*), 6.83 (t, 1H, *Ar-H*).^13^C NMR (DMSO-d6/TMS, ppm): δ = 162.73 (N-*C*=O), 161.65 (*Ar*-OH), 160.38 (*Ar*-OH), 146.59 (*C*H = N), 145.95 (*Ar*-H), 139.80, 139.15 (*Ar*-NO_2_), 139.15 (*Ar*-NO_2_), 134.59, 128.19, 126.82, 126.37, 125.07, 124.76, 122.19, 119.41, 118.10, 117.34, 116.37, 114.99 and 113.12 (*Ar*-H), 66.36 (*C*H quinazoline ring). X-ray quality crystals were grown from CH_3_CN.

### Crystallography

Diffraction data were collected on a Bruker SMART Apex II CCD area-detector diffractometer (graphite-monochromated Mo-Kα radiation, λ = 0.71073Å) at 296(2) K. The orientation matrix, unit-cell refinement, and data reduction were all handled by the Apex2 software (SAINT integration, SADABS absorption correction)^36^. The structure was solved using direct methods in the program SHELXS-97 and was refined by the full matrix least-squares method on F2 with SHELXL-2013^37^. The non-hydrogen atoms were refined anisotropically, and the C-bound hydrogen atoms were added geometrically and refined with a riding model. The N- and O-bound H atoms were located in a difference Fourier map and refined freely. Molecular graphics were prepared using XSEED software26^37^.

### Anticancer activity of quinazoline Schiff bases

#### Culture

The human breast cancer MCF-7, human normal breast cells MCF-10A and human normal hepatic cells WRL-68 were obtained from American Type Culture Collection (ATCC) and cultured in RPMI-1640 medium that was supplemented with 10% fetal bovine serum (FBS). Cells were cultured in tissue culture flasks (Corning, USA) and were kept in an incubator under standard conditions (37 °C in a humidified atmosphere with 5% CO2). All cells were grown to approximately 70% confluence in corning flasks.

### Cell viability MTT Assay

The cytotoxic effect of the compounds were assessed by MTT cell viability assay against MCF-7, MDA-MB-231, MCF-10A and WRL-68 cell lines. Cell lines were seeded into 96-well transparent flat bottom plates (Greiner Bio-One) at a concentration of 5 × 10^5^ cells/well for different treatment periods (24, 48, and 72 hours). On the next day, the cells were treated with different concentrations of compounds (1.563, 3.125, 6.25, 12.5, 25, and 50, 100 *μ*g/mL). After 24, 48 and 72 hours of incubation, 20 μl of a solution containing 4,5-dimethylthiazol-2-yl-2,5-diphenyltetrazoliumbromide (MTT, Santa Cruz) at a concentration of 5 mg/mL was added to each well of the microplate. After 4 hours, media with MTT reagent was removed before adding 100 *μ*L of dimethylsulfoxide (DMSO) to solubilize the formazan crystals. The optical density was measured with an ELISA microplate reader at an absorbance of 570 nm. The assay was performed in triplicate to calculate the half maximal inhibitory concentration (IC_50_) value. MCF-7 cells were also treated with a standard drug, doxorubicin, as a positive control.

### LDH Assay

The cytotoxicity of the compounds was also determined by the lactate dehydrogenase (LDH) release assay on MCF-7 cells. LDH release in the medium is due to the loss of membrane integrity either due to apoptosis or necrosis. Briefly, MCF-7 cells were treated with different dosages of the compounds for 48 hours. Then, the supernatant of the treated cells was relocated into 96-well plates, and 100 μl of the LDH reaction solution (PierceTM LDH Cytotoxicity Assay Kit, Thermo Scientific™, Pittsburgh, PA) was added for 30 minutes. Finally, the intensity of red color in the samples indicating the LDH activity was measured at 490 nm using a Tecan Infinite®200 Pro (Tecan, Männedorf, Switzerland) microplate reader. LDH release increased in a dose-dependent manner in A or B treated MCF-7 cells compared with untreated cells. The values are represented as the means ± SD of three separated experiments.

### Acridine Orange and Propidium Iodide Staining

Dual-Fluorescence staining using acridine orange (AO) and propidium iodide (PI) is a nuclear staining method to assess apoptotic cell morphology. AO is able to permeate into both live and dead cells to stain all nucleated cells and excite green florescence, while PI only enters dead cells with damaged membranes and generates red florescence. Thus, late apoptotic and necrotic cells take up both stains. Briefly, MCF-7 cells were treated with IC_50_ concentration of the compounds for 48, and 72 hours treatment periods. In this experiment, untreated MCF-7 cells were also stained as a negative control. After each period of treatment, cells were washed twice using PBS to remove the remaining media. Then, cells were stained by adding equal volumes of AO and PI (10 *μ*g/mL) (Sigma, UK). The cell suspension (10 μL) was placed onto a glass slide and morphologically assessed with a fluorescence microscope within 30 minutes before the fluorescence began to fade. The criteria for identification were as follows: (a) green intact nucleus, viable cells; (b) dense green areas of chromatin condensation in the nucleus, early apoptosis; (c) dense orange areas of chromatin condensation, late apoptosis; and (d) orange intact nucleus, secondary necrosis^30^.

### Reactive Oxygen Species Assay

A ROS assay was carried out to determine the influence of compounds on the production of ROS levels in A- and B- treated MCF-7 cells. ROS activity was measured by a Cellomics Oxidative Stress 1 HCS Reagent Kit (Thermo Scientific, Pittsburgh, PA), according to the manufacturer’s protocols. Briefly, 1 × 10^4^ cells per well were seeded into 96-well plates and incubated overnight at 37 °C under conditions of 5% CO2. The cells were then treated with different concentrations of the compounds. After 24 hours, dihydroethidium (DHE) dye was added into live culture for 30 minutes. Cells were fixed and washed with wash buffer. The DHE dye probe is oxidized to ethidium in the presence of superoxides. The fluorescence intensity was measured using a fluorescent plate reader at an excitation wavelength of 520 nm and an emission wavelength of 620 nm. The values are represented as the means ± SD of three sets of experiments.

### Multiple Cytotoxicity Assay

A Cellomics multiparameter cytotoxicity 3 kit (Thermo Fisher Scientific, Waltham, MA, USA) was applied as previously described^48^,^49^. This kit simultaneously measures some independent parameters in the same cell, including changes in mitochondrial membrane potential (MMP), cytochrome c release, and cell membrane permeability. Briefly, 1 × 10^4^ cells per well were seeded into a 96-well plate. After 24 hours, cells were treated with different concentrations of A and B or dimethylsulfoxide (negative control). Next, MMP dye and cell permeability dye were added to live cells and incubated for 30 minutes at 37 °C. Cells were then fixed, permeabilized, and blocked with 1X blocking buffer prior to probing with primary cytochrome *c* primary antibody and secondary DyLight 649 conjugated goat antimouse immunoglobulin G for 1 hour each. Then, Hoechst 33342 was added into the staining solution to stain the nucleus. Finally, the plates were analyzed using ArrayScan high content screening system (Thermo Fisher Scientific). The ArrayScan high content screening system is a computerized automated fluorescence imaging microscope that automatically identifies stained cells and reports the intensity and distribution of fluorescence in individual cells. In each well, 1,000 cells were analyzed. Images were acquired for each fluorescence channel using suitable filters. Images and data regarding intensity and texture of the fluorescence within each cell, as well as the average fluorescence of the cell population within the well, were stored in a Microsoft SQL database for easy retrieval. Data were analyzed with ArrayScan II Data Acquisition and Data Viewer version 3.0 software (Thermo Fisher Scientific). The values are represented as the means ± SD of three sets of experiments.

### Caspase-3/7,-8 and -9 Activity Bioluminescent Assays

Caspase-Glo® 3/7, 8 and 9 kits (Promega, Madison, WI) were applied to measure the activation of caspase-3/7, -8 and -9 according to the manufacturer’s protocols^50^. Briefly, cells were treated with A and B at IC_50_ concentrations for 24 hours of treatment in a time-dependent manner (6, 12, 18, 24 and 30 hours) and exposed to caspase-Glo reagent (100 μL) for 30 minutes. Finally, the caspase activity was determined as the degree of aminoluciferin-labelled synthetic tetrapeptide cleavage and luciferase enzyme substrate release from the cells as operated by a Tecan Infinite®200 Pro microplate reader (Tecan, Männedorf, Switzerland). The values are represented as the means ± SD of three sets of experiments.

### Western Blotting

Western blotting was used to examine the expression of apoptosis-related proteins, including caspase-9, -7 and Bid. MCF-7 cells were treated with A and B in a concentration-dependent manner for 24 hours. Untreated cells served as the negative control. The total protein content in the cells was extracted with cell lysis buffer (50 mM Tris-HCl pH 8.0, 120 mM NaCl, 0.5% NP-40, 1 mM phenylmethylsulfonyl fluoride); 40 μg of protein extract was separated by 10% sodium dodecyl sulfate polyacrylamide gel electrophoresis and then transferred to a polyvinylidene difluoride membrane (Bio-Rad, Hercules, CA, USA) using a semidry transfer unit (TE 70X, Hoefer Inc., Holliston, MA, USA), blocked with 5% nonfat milk in TBS-Tween buffer (0.12 M Tris-base, 1.5 M NaCl, 0.1% Tween 20) for one hour at room temperature, and incubated overnight at 4 °C with the appropriate primary antibodies, that is, β-actin (1:5,000), caspase-9 (1:1,000), caspase-7 (1:1,000), and Bid (1:1,000) purchased from Santa Cruz Biotechnology Inc. (Santa Cruz, CA, USA). This was followed by incubation with alkaline phosphatase-conjugated goat anti-mouse and goat anti-rabbit secondary antibodies (i-DNA, Promega, Madison, USA) for 30 minutes and subsequent washing in Tris-buffered saline with Tween 20 for 10 minutes. The blots were then developed using BCIP®/NBT solution (Santa Cruz Biotechnology Inc.) during a period of 5–30 minutes to detect the target protein band as a precipitated dark-blue color^44^,^48^.

### NF-κB activation assay

Briefly, MCF-7 cells (1.0 × 10^4^) were seeded into a 96-well plate. On the next day, the cells were treated with a different dosage of A and B for 3h ours and stimulated with 1 ng/mL tumor necrosis factor alpha (TNF-α) for 30 minutes. Medium was then removed and the cells were fixed and stained with a nuclear factor kappa B (NF-κB) activation kit from Thermo Fisher Scientific according to the manufacturer’s instructions. The plate was analyzed by an ArrayScan high content screening reader. Finally, the intensity ratio of cytoplasmic and nuclear NF-κB was determined using Cytoplasm to Nucleus Translocation BioApplication software (Thermo Fisher Scientific). The average intensity of 200 objects (cells) per well was quantified. All values are mean of three experiments. The ratios were compared between TNF-α-stimulated, treated, and untreated cells^30^,^48^.

### Acute toxicity study

The acute toxicity study was conducted according to the OECD protocol^51^.Twelve ICR female mice were used for the acute toxicity study to evaluate the toxicity of the compounds. All experimental protocols were approved by the ethics committee of the Faculty of Medicine, University of Malaya, Malaysia (Ethic No.PM/27/07/2010/MAA (R)). The methods were carried out in accordance with the National Academy of Science’s Guide for the Care and Use of Laboratory Animals^52^. Two groups of animals was considered: the normal control group, which received only vehicle, and the treated groups, which received a 250 mg/kg dosage of the compounds^30^,^52^. Prior to the experiment, all mice were fasted for 24 hours. After treatment, the animals were observed for the first 30 minutes and 4–5 times at intervals of 48 hours to discern any signs of abnormality. After 14 days, the animals were sacrificed by an overdose of xylazine and ketamine anesthesia. Blood samples were then collected for serum biochemical examination. In addition, kidney and liver histological analysis was performed using hematoxylin and eosin (H&E) staining. All values are mean of three experiments.

### Statistical Analysis

Experimental values are presented as the means ± standard deviation (SD) for 3 independent experiments. Analysis of variance (ANOVA) was performed using GraphPad Prism 5 software. Statistical significance was defined when P < 0.05.

## Additional Information

**How to cite this article**: Zahedifard, M. *et al.* Synthesis, characterization and apoptotic activity of quinazolinone Schiff base derivatives toward MCF-7 cells via intrinsic and extrinsic apoptosis pathways. *Sci. Rep.* **5**, 11544; doi: 10.1038/srep11544 (2015).

## Figures and Tables

**Table 1:** X-ray crystallographic data table for compound (B).

Identification code	Compound (B)
**Empirical formula**	C_23_H_18_N_6_O_7_
**Formula weight**	490.43
**Temperature/K**	296(2)
**Crystal system**	monoclinic
**Space group**	P2_1_/n
**a/Å**	10.757(2)
**b/Å**	15.543(4)
**c/Å**	14.063(3)
**α/°**	90.00
**β/°**	92.781(4)
**γ/°**	90.00
**Volume/Å**^**3**^	2348.5(9)
**Z**	4
**ρ**_**calc**_**g/cm**^3^	1.387
**μ/mm**^**-1**^	0.106
**F(000)**	1016.0
**Crystal size/mm**^3^	0.17 × 0.13 × 0.09
**Radiation**	MoKα (λ = 0.71073)
**2Θ range for data collection/°**	4.6 to 52.78
**Index ranges**	−13 ≤ h ≤ 11, −17 ≤ k ≤ 19, −15 ≤ l ≤ 17
**Reflections collected**	12436
**Independent reflections**	4763 [R_int_ = 0.0271, R_sigma_ = 0.0349]
**Data/restraints/parameters**	4763/6/338
**Goodness-of-fit on F**^**2**^	1.008
**Final R indexes [I>=2σ (I)]**	R_1_ = 0.0435, wR_2_ = 0.1106
**Final R indexes [all data]**	R_1_ = 0.0791, wR_2_ = 0.1315
**Largest diff. peak/hole / e Å**^**-3**^	0.17/−0.15

Crystal Data for compound (B) C_23_H_18_N_6_O_7_ (*M* =490.43 g/mol): monoclinic, space group P2_1_/n (no. 14), *a* = 10.757(2) Å, *b* = 15.543 (4) Å, *c* = 14.063 (3) Å, *β* = 92.781 (4)°, *V* = 2348.5 (9) Å^3^, *Z* = 4, *T* = 296 (2) K, μ(MoKα) = 0.106 mm^-1^, *Dcalc* = 1.387 g/cm^3^, 12436 reflections measured (4.6° ≤ 2Θ ≤ 52.78°), 4763 unique (*R*_int_ = 0.0271, R_sigma_ = 0.0349) which were used in all calculations. The final *R*_1_ was 0.0435 (>2sigma(I)) and the *wR*_2_ was 0.1315 (all data).

**Table 2:** IC_50_ values of compounds against MCF-7, MCF-10A and WRL68 cell lines.

Compound	Cell line	Classification	IC_50_ (μg/ml)		
			**24 h**	**48 h**	**72 h**
**(A)**	MCF-7 MDA-MB-231	Breast cancer cells	7.42 ± 0.423 50<	3.76 ± 0.251 50<	3.27 ± 0.171 50<
	MCF-10A WRL-68	Normal breast cells Normal hepatic cells	25< 40<	25< 40 <	25< 40<
**(B)**	MCF-7 MDA-MB-231	Breast cancer cells	10.67 ± 0.551 50<	5.87 ± 0.401 50<	4.36 ± 0.219 50<
	MCF-10A WRL-68	Normal breast cells Normal hepatic cells	25< 40<	25< 40 <	25< 40<
**Doxorubicin**	**MCF-7**	**Breast cancer cells**	**2.43** **±** **0.24**	**2.28** **±** **0.33**	**2.08** **±** **0.16**

**Table 3:** Effects of 250 mg/kg A and B on the liver function test.

Groups	Total protein (g/L)	Albumin (g/L)	Globulin (g/L)	AP (IU/L)	ALT (IU/L)	AST (IU/L)	GGT (IU/L)
**Vehicle**	52.0 ± 1.5	12.4 ± 0.65	51.4 ± 1.1	85.3 ± 3.2	58.9 ± 5.4	251 ± 8.4	3.5 ± 0.1
**1 (250 mg/kg) 2 (250 mg/kg)**	52 ± 1.5 59 ± 1.0	10.5 ± 0.62 11.2 ± 0.43	51.3 ± 1.2 50.6 ± 1.4	88.6 ± 2.8 90.2 ± 3.1	62.2 ± 4.3 60.0 ± 4.4	265 ± 6.5 244 ± 5.9	3.1 ± 0.08 3.0 ± 0.05

Values are expressed as the means ± S.E.M. There were no statistically significant differences between the measurements of different groups. Significance was set at *P* < 0.05.

**Table 4:** Effects of 250 mg/kg A and B on the renal function test.

Groups	Sodium (mmo/L)	Potassium (mmol/L)	Chloride (mmol/L)	CO_2_(mmol/L)	Anion gap (mmol/L)	Urea (mmol/L)
**Vehicle**	145 ± 0.91	8.7 ± 0.15	109.4 ± 0.32	20.5 ± 0.34	27.0 ± 0.6	6.5 ± 0.7
**1 (250 mg/kg)**	148.3 ± 0.68	9.0 ± 0.12	108.5 ± 0.36	23.5 ± 0.78	25.9 ± 0.7	6.8 ± 0.4
**2 (250 mg/kg)**	152.9 ± 0.66	8.2 ± 0.1	113.8 ± 0.38	18.2 ± 0.28	23.2 ± 0.4	7.9 ± 0.3

Values are expressed as the means ± S.E.M. There were no statistically significant differences between the measurements of different groups. Significance was set at *P*  < 0.05.
